# Medical students’ perception of online intensive pediatric review: an experimental cross-sectional study

**DOI:** 10.1186/s12909-023-04757-5

**Published:** 2023-10-19

**Authors:** Sirikarn Tangcheewinsirikul, Preyanit Takkinsatian, Patcha Yenjabog, Ornatcha Sirimongkolchaiyakul, Pathaporn Prempraparn

**Affiliations:** Department of Pediatrics, Faculty of Medicine Vajira Hospital, Navamindradhiraj University, 681 Samsen Road, Dusit, Bangkok 10300 Thailand

**Keywords:** Online education, Perception, Medical students, Undergraduate students, Pediatrics

## Abstract

**Background:**

Despite regular pediatric education, pediatric instructors regularly provide an on-site intensive pediatric review course (IPR) as per medical students (MS)’ request, to summarize pediatric knowledge for fifth-year MS in preparation for their externship. However, considering the coronavirus disease 2019 (COVID-19) pandemic restrictions (e.g., social distancing), an online intensive pediatric review (OIPR) is required instead. Unfortunately, the relationship between MS’ perception and outcome of OIPR remains unclear.

**Methods:**

We developed the OIPR and an online mock pediatric examination (OMPE), aligning it with the essential pediatric components of the Medical Council curriculum. The OIPR comprised of two parts: self-paced online learning and in-class online discussions. The self-paced online learning materials were electronically distributed via Google Classroom to MS ten days priors to the one-day course, which included a pretest, in-class online discussions, posttest, and satisfactory survey. The constructed and validated satisfactory survey was categorized into two parts: demographic data and self-perceived satisfaction with OIPR. For data collection, an anonymous self-administered survey was used and was distributed to MS in April 2022. These data were then analyzed by Wilcoxon signed rank test.

**Results:**

Of the 80 eligible fifth-year MS, 45 agreed to participate (56.3%), of which 24 (53.3%) were females. The mean ± standard deviation (SD) of MS’ age was 23 ± 0.6 years. All (100%) concurred that OIPR is beneficial and recommended it to junior students who were planning to take the examination. The mean ± SD of OMPE significantly increased, from 20.9 ± 3.8 to 22.9 ± 3.3 (*p* = 0.001).

**Conclusion:**

During the peak of the COVID-19 pandemic, which required social distancing, OIPR has helped MS summarize and enhance their knowledge in preparation for externship and the examination.

## Background

The coronavirus disease 2019 (COVID-19) is a serious communicable disease that has affected not only physical health but also economic activities, society, and education in various countries worldwide [1]. Its outbreak has led to difficult situations for many countries, including Thailand, and policies and procedures for restricting close contact and maintaining a distance have been established for infection control. The implementation of the regulation has resulted in limited social activities, including the teaching and learning of medical students (MS) [2], especially in the clinical clerkship period. These restrictions may affect the experience and competence of MS in patient encounters as well as their proficiency level for the National License Examination (NLE).

The Diploma of Medicine in Thailand is divided into six years of curriculum. All MS need to be assessed by NLE according to the criteria for assessing knowledge and competence for obtaining a medical license (2012) from the Medical Council of Thailand [3]. These criteria consist of three steps, namely, assessment of fundamental health science knowledge (NL1), assessment of clinical knowledge (NL2), and the objective structured clinical examination (OSCE), which are conducted in the third, fifth, and sixth year of MS, respectively. Before the final year, the pediatric staff in our faculty has been providing an on-site intensive pediatric review course (IPR) as requested by MS, to summarize and enhance their knowledge in preparation for externship and the examination. However, the teaching methods have been substituted to online learning or teleconference because of the epidemic situation and the regulation of limited group activities. In this study, we aimed to evaluate fifth-year MS’ perception of online intensive pediatric review (OIPR) and the outcome of OIPR by using self-administered online survey and pre–post online mock pediatric examination (OMPE).

## Methods

The Ethics Committee for Research in Humans reviewed and approved our study protocol (approval No.: COA 054/2565). Due to the time limitation of 30 min for the test, we engaged in brainstorming sessions and finally opted to construct a 30-items multiple choice questions, known as OMPE, which comprehensively covered all essential topics in pediatric curriculum of our faculty and the Medical Council of Thailand. The development of OMPE was undertaken by a pediatric working group comprising five pediatricians in our faculty. The validity of OMPE was evaluated from five pediatric experts using Index of Item-objective congruence (IOC) [4], indicating a good content validity ranging from 0.6–1. Furthermore, the OMPE demonstrated a satisfactory reliability of 0.75, as indicated by the Kuder-Richardson 20 coefficient. All MS provided informed consent to participate in the study. An author who was not involved in the OIPR was assigned to distribute OMPE to MS before and after the in-class online discussion.

**Fig. 1:**
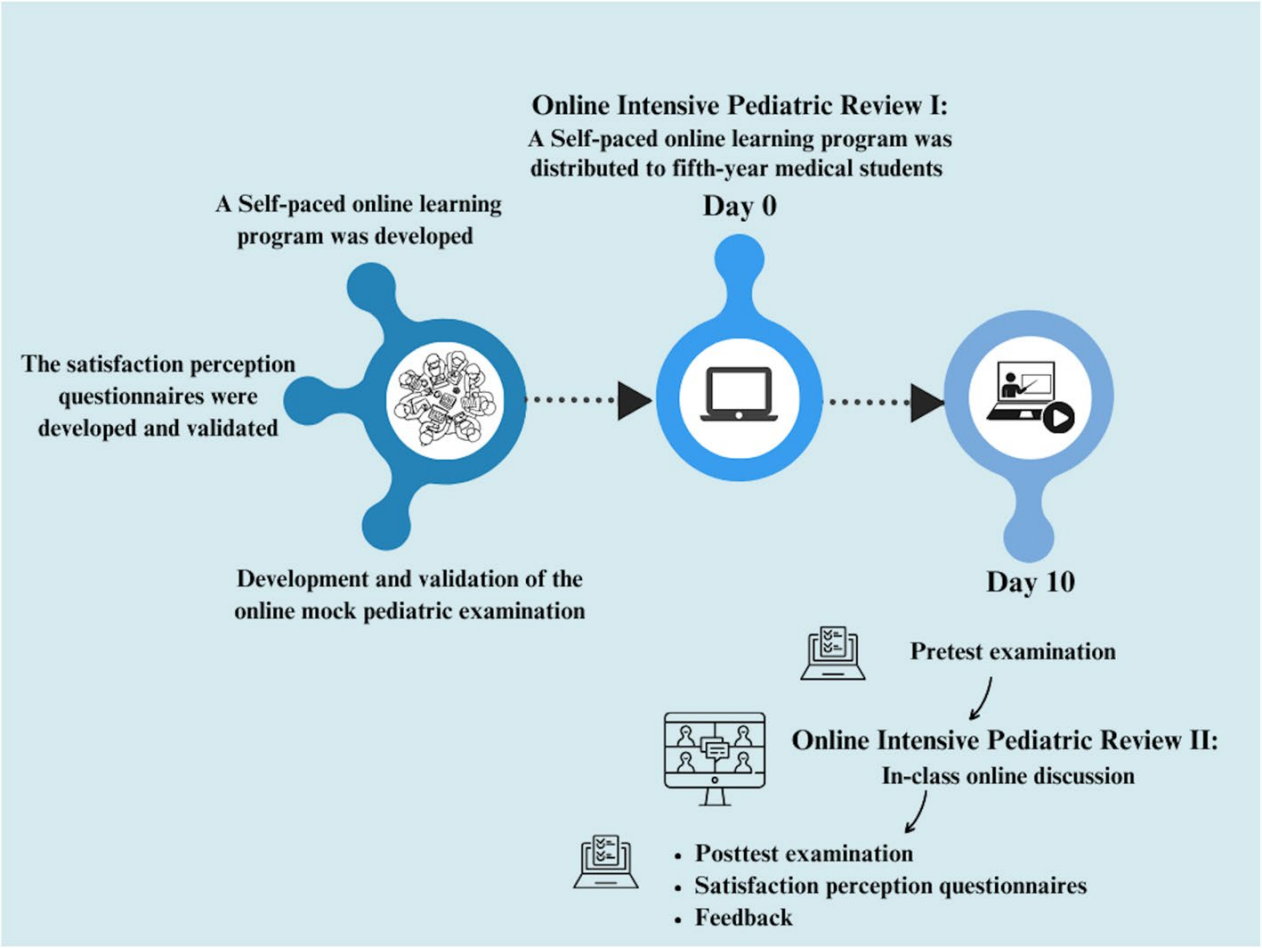
Timeline of the online intensive pediatric review and questionnaires development

The OIPR was designed by the faculty to summarize and enhance MS’ knowledge and practice and discuss question banks, which include a class-sharing opinion. A pediatric working group developed the OIPR, which is divided into two parts: self-paced online learning and in-class online discussion. The self-paced online learning module offered eight hours of content through five videos, covering the essential part of allergy, cardiology, dermatology, gastroenterology, hematology-oncology, infectious diseases, neonatology, nephrology, neurology, nutrition, and rheumatology which were in accordance with the curriculum outlined by the Medical Council of Thailand. The program was delivered to MS through Google Classroom, which restricted access exclusively to faculty members in order to content and patient privacy. All pediatric working members agreed to provide learning slides and handouts as graphically as possible, and self-pace online learning which was a part of OIPR and handouts were electronically distributed to MS 10 days prior the in-class online discussion (Fig. 1). The in-class online discussion was conducted over the course of one day, covering the essential part aligned with the pediatric curriculum contents; it primarily consisted of knowledge sharing, group discussions, and question bank practice.

After the posttest, a self-administered structured satisfaction online questionnaire was distributed to MS who indicated that they had completed the self-pace online learning via a QR code. Informed consent was inferred when students completed and returned the questionnaires. The first section of the questionnaire covered MS’ demographic information, previous grade point average (GPA) in Pediatrics, and accumulated GPA (GPAx). The second section included satisfaction perception questions, which were answered by a 5-point Likert scale, with an additional space for free-text comments at the later part. The 5-point Likert scale is described as follows: 1, strongly disagree; 2, disagree; 3, neutral; 4, agree; and 5, strongly agree. The initial version of the self-administered online questionnaire consisted of 22 questions aimed at assessing medical students’ perception of OIPR during the COVID-19 pandemic. These questions were developed based on a literature review and a focus group. The questionnaires were then distributed to three medical educational experts for validity assessment using the item-content validity index (i-CVI). One question was removed due to an i-CVI of 0.3, indicating inadequate validity, while the remaining 21 questions underwent minor revision to enhance precision. The revised self-administered online questionnaire was subsequently evaluated by the same experts, revealing i-CVI scores ranging from 0.67 to 1.0, with an overall content validity for the scale (S-CVI) of 0.87. Additionally, the revised version was distributed to a separate group of 40 MS participants to assess reliability, demonstrating a Cronbach’s Alpha coefficient of 0.96. The data were privately collected online using the free Google Forms survey administration software. Throughout the study, every MS’ anonymity and confidentiality were maintained.

Continuous data are reported as means and standard deviations (SD), or for non-normally distributed variables, as medians and interquartile ranges (IQR). Categorical data are presented as numbers and percentages. For independent population analyses, non-parametric data were compared using the Wilcoxon-signed rank test. All statistical data were analyzed using the IBM SPSS version 28.0 for Windows (IBM Corp., Armonk, NY, USA), and *p*-values of less than 0.05 were considered statistically significant.

## Results

Of the 80 fifth-year MS, 45 agreed to participate (56.3%), with 24 females and 21 males. Table 1 summarizes the demographic data. Their mean age was 23.1 ± 0.6 years, and the median (IQR) GPAx was 3.4 (3.2, 3.6), with approximately two-fifths of the students reported A and B + as their Pediatrics GPA.

**Table 1:** Demographic and clinical characteristics of the medical students

**Variable**		**N (%)**
Age (year), mean ± SD		23.1 ± 0.6
Gender	Male	21 (46.7)
	Female	24 (53.3)
GPAx, median (IQR)		3.4 (3.2, 3.6)
Pediatric GPA	A	9 (20)
	B +	9 (20)
	B	5 (11.1)
	C +	7 (15.6)
	C	4 (8.9)
	N/A	11 (24.4)

Abbreviations: *GPA* Grade point average, *GPAx* Accumulated grade point average, *IQR* Interquartile range, *SD* Standard deviation

**Fig. 2:**
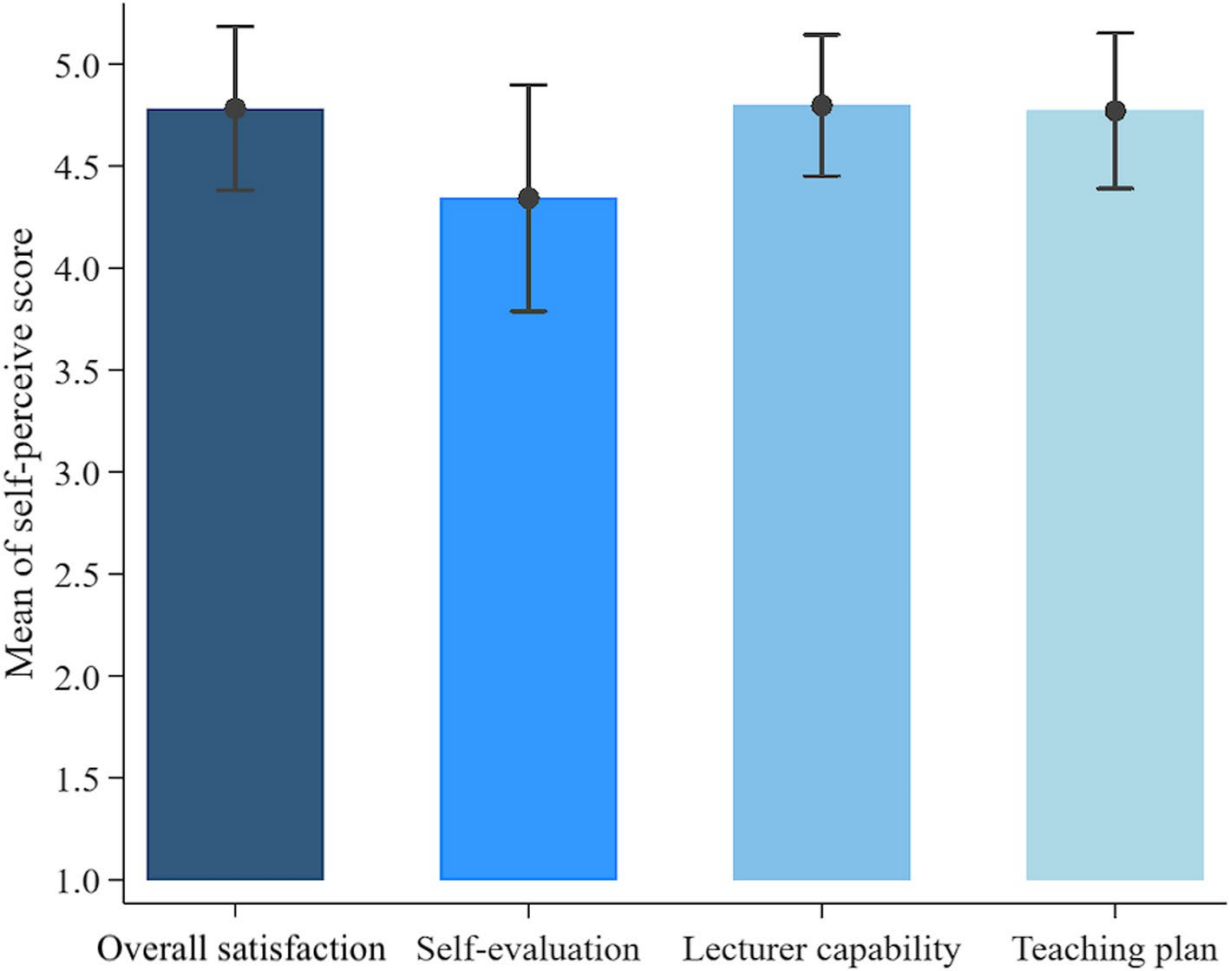
Self-perceived satisfaction among four domains

The self-perceived satisfaction with OIPR by MS was categorized into four sections: overall course satisfaction factors, self-preparedness evaluation, instructors’ capability skill, and course curriculum. Table 2 lists the results from each section. Overall, the MS were satisfied, with strong agreement in all domains, including the topic, instructors, instructor skill in summarizing materials, question banks in-class practice, and discussion, with an average of 4.8 ± 0.4. Compared with other domains, the MS self-evaluation domain obtained a rather low mean score (Fig. 2). Over one-third of MS reported having doubts about their prior course knowledge and confidence in pediatrics, accounting for 4.0 ± 0.9, followed by confidence in pediatrics competence (4.2 ± 0.9).

**Table 2:** Perception of medical students among online intensive pediatric review course

Topic	Score, n (%)				
	**1**	**2**	**3**	**4**	**5**
**Overall course satisfied factor**					
1. Topic	-	-	-	20 (22.2)	35 (77.8)
2. Lecturer	-	-	-	11 (24.4)	34 (75.6)
3. Summary skill of Lecturer	-	-	1 (2.2)	8 (17.8)	36 (80.0)
4. Question banks in-class practice	-	-	1 (2.2)	7 (15.6)	37 (82.2)
5. Question banks discussion	-	-	-	9 (20)	36 (80)
**Self-assessment domain**					
7. Prior course confidence	-	-	14 (31.1)	9 (20)	22 (48.9)
8. Prior course knowledge	-	-	17 (37.8)	10 (22.2)	18 (40.0)
9. Post course pediatric developmental skill and knowledge	-	-	-	16 (35.6)	29 (64.4)
10. Post course knowledge	-	-	-	23 (51.1)	22 (48.9)
11. Post course confidence in NLE	-	-	3 (6.7)	22 (48.9)	20 (44.4)
**Instructor domain**					
12. Instructor teaching skill	-	-	-	11 (24.4)	34 (75.6)
13. Instructors’ preparedness	-	-	-	7 (15.6)	38 (84.4)
14. Instructors’ ability to stimulate class learning environment	-	-	-	10 (22.2)	35 (77.8)
16. Timely management of instructors	-	-	1 (2.2)	11 (24.4)	33 (73.3)
17. Instructors provided response and feedback	-	-	-	9 (20)	36 (80)
18. Helpful suggestions from instructors	-	-	-	7 (15.6)	38 (84.4)
19. Proper educational media used	-	-	-	7 (15.6)	38 (84.4)
**Course curriculum domain**					
20. Constructed and valid course curriculum	-	-	-	9 (20)	36 (80)
21. Well prepare teaching curriculum	-	-	-	8 (17.8)	37 (82.2)
22. Proper quantity of curriculum	-	-	-	14 (31.1)	31 (68.9)
23. Amenities; Wi-Fi, Media, Slide, Handout	-	-	-	15 (33.3)	30 (66.7)

Abbreviation: *NLE* National license examination

The instructors’ pedagogical skill domain assessed by MS obtained a mean score as high as 4.8 ± 0.3. Most of the MS were generally satisfied with the instructors’ teaching capability, preparedness for the course, promptness in responding to inquiries, and level of helpfulness in providing guidance. One MS rating, three out of five, was given for the instructors’ timely class management, and a free-text comment proposing to increase extra class time was mentioned.

Furthermore, the course curriculum domain score was related to the lecturer's pedagogical skill and feedback, with a mean score of 4.8 ± 0.4. Wi-Fi and other infrastructures that are helpful during restricted in-person contact were used in relation to the online teaching course. Nevertheless, the overall satisfaction score was as high as 4.7 ± 0.5.

While the MS answered the questions with a 5-point Likert scale, they mentioned many free-text comments to the OIPR…

*“I really appreciate the pedagogical skill of the pediatric lecturers.”*

*“As I rotated in Pediatric ward in the first turn of my fifth-year medical student, this is a great opportunity to summarize and emphasize the critical point in Pediatrics.”*

*“This course has the discussion part that covers both knowledge and question banks, which guides me in focusing on the should-know and must-know aspects of pediatrics.”*

*“The internet quality is interrupted; however, the provided electronic handouts are superb.”*

*“I hope that this course will be continued.”*

**Fig. 3:**
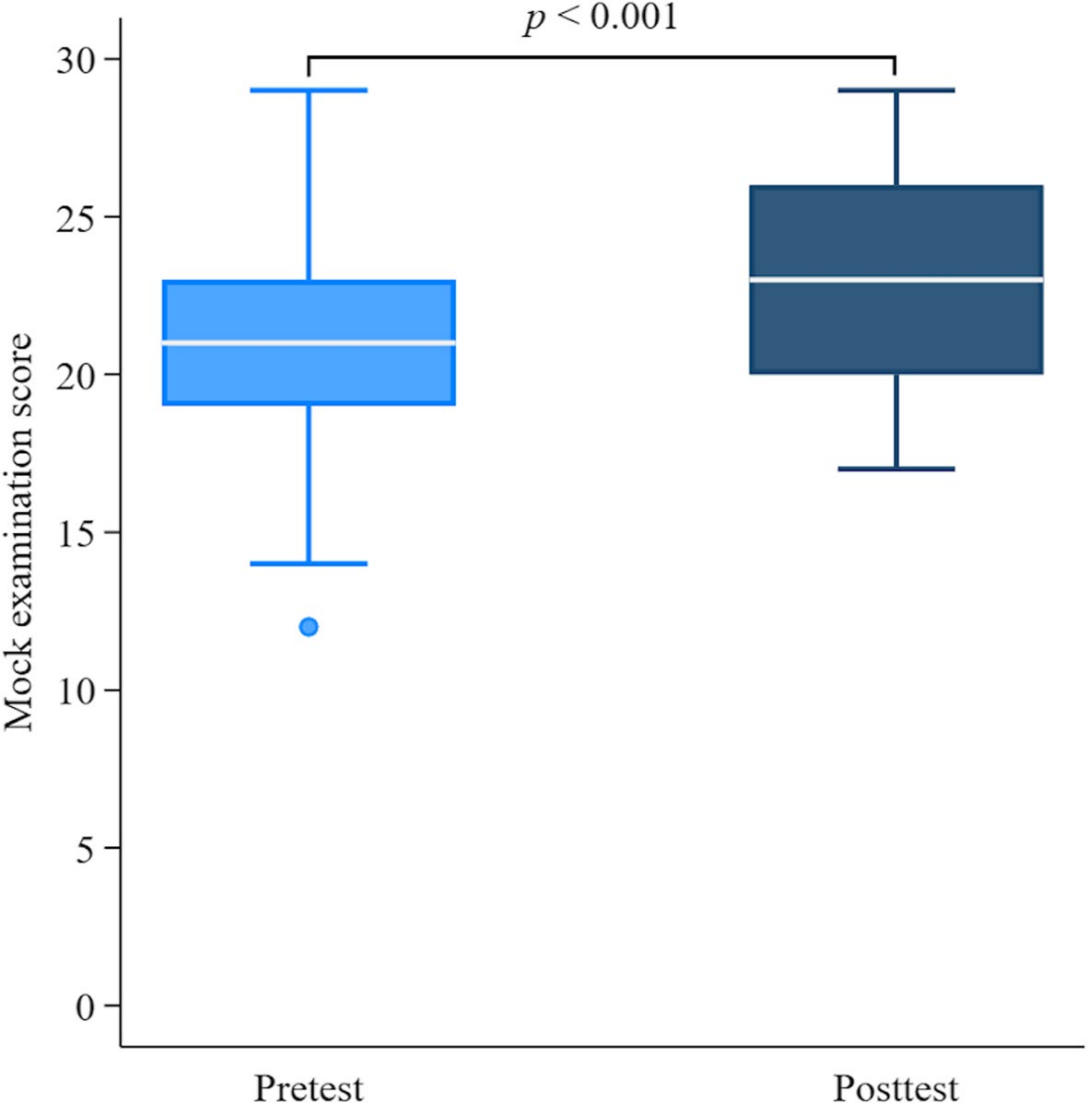
Pre- and post-online intensive pediatric review II information among medical students

For the study’s secondary outcome, we evaluated the changes between pre- and post-in-class online discussion by OMPE. All MS participated in the pre- and posttest within the same day, except for 2 individuals who completed the assessment after, but within 14 days. The mean score of pretests was 20.9 ± 3.8, with a minimum of 12 and a maximum of 29 (Table 3). Following the in-class online discussion, the MS were re-evaluated using the same multiple-choice questions. As shown in Fig. 3, the mean score of posttest increased to 22.9 ± 3.3 (*p* = 0.001). Ultimately, all MS (100%) concurred that OIPR is useful and expressed their recommendation for juniors planning to take the NL examination next year.

**Table 3:** Pre- and post-online intensive pediatric review information among medical students. A *p*-value < 0.05 indicates statistical significance

Factor		Pre OIPR-II	Post OIPR-II	*X (95 CI)*	*p*
Perception of knowledge, mean ± SD		4.0 ± 0.9	4.5 ± 0.5	0.5 (0.2–0.7)	< 0.001*
Confidence for NL, mean ± SD		4.2 ± 0.9	4.4 ± 0.6	0.2 (-0.5–0.4)	0.107
Mock examination					
	Min	12	17		
	Max	29	29		
	Mean ± SD	20.9 ± 3.8	22.9 ± 3.3	2.0 (1.0–3.1)	0.001*

Abbreviation: *CI* Confident interval, *NL* National license examination, *OIPR* Online intensive pediatric review, *SD* Standard deviation

## Discussion

As a result of the nationwide measures for the COVID-19 pandemic, social activities such as in-person meetings are restricted, thereby affecting the quality of education, including that of medical education. Although virtual education was adopted as an alternative for in-person education in many departments, its quality and efficacy have remained undetermined [5]. Students have different perceptions toward online education. In British’s perspective of online education [6], Dose S et al. reported active learners have greater flexibility and properly self-managed time. However, Mortazavi F et al. reported that students may be exposed to family distraction and limited online infrastructure [7]. The MS in our center have associated perceptions on online education, reflecting the optimistic feedback in flexible time management, while one have revealed that drawbacks from online infrastructure have effects on OIPR quality, consistent with the reports conducted in the UK and Egypt [6, 8].

Our OIPR approach was brainstormed and eventually divided into two parts: self-paced learning and in-class online discussion. This active learning style was inspired by the flipped classroom model, which has been effective in medical learning studies [9–11]. Despite the learning style debate [12], a meta-analysis of 225 studies found that active learning, which includes in-class discussion, improves learner concept inventories and exam performance [13] compared with the traditional learning styles, which have demonstrated an exam failure rate that is 1.5 times that of active learning [14]. While infectious diseases have an impact on in-person contact, OIPR can provide MS inquiries. In France, OIPR, which includes a discussion with the instructors and provides quality feedback, promotes learning and understanding for learners [15]. This finding might explain the score of 5 in overall course satisfaction in more than 80% of MS, particularly in the instructor's skill, in-class practice, and discussion.

Our study demonstrated a high satisfaction score of 5 regarding course amenities, accounting for 30 (66.7%), specifically the utilization of media, slides, and handouts to enhance in-class focus and facilitate comprehension. To optimize the learning benefits for our MS, our working group has discussed about and concentrated on the importance of slide and handout formats. The group finally decided to make the slide and handout as graphically as possible and distributed them to MS prior the OIPR. These tools possibly augmented student engagement more than simply reading the material, and achieved a higher postlearning test score than the traditional handouts, as reported by Junhasavasdikul et al. [16] In addition, Wongkietkachorn A et al. found that 83.6% of MS may decrease class engagement and increase class nonattendance in the absence of handouts [17]; however, they found no relationship between GPA and attitude in learning with limited handouts [17].

This study aims to address the perceptions of MS who are the primary stakeholders of the OIPR. Most of the MS strongly agreed with the utilization of OIPR, obtaining an overall satisfactory rate of 80% and above, especially in terms of the instructors’ skill of summarization, question banks in-class practice, and discussion. In many medical schools from various countries, the use of question banks and other online resources has been useful for MS in shaping points to consider in a broad range of knowledge [6, 18]. Although quantity evaluation in our study was conducted through pre- and post-OMPE. This allowed us to assess the impact of OIPR, specifically the increase in OMPE scores after the in-class online discussions, as an indicator of its potential effectiveness in evaluating MS short-term achievement.

Our study has several limitations. First, this single-center study included only a small number of MS, without power calculation, thereby possibly reducing the outcome difference and affecting the statistical significance. Second, the questionnaire did not include all curricula in detail, except for pediatrics, thereby not reflecting the overall percentage pass in the NL examination of our faculty. Furthermore, to account for potential outcome variations due to different timeframes, in-class online discussion, pre- and post-OMPE were mainly completed within a single day, considering its potential impact on retention rate. The result may predominantly reflect short-term memory only. Additionally, the self-paced online learning materials distributed to MS through Google classroom limited the capability to monitor individual completion in term of the timing of online learning; it solely recorded the name of views, indicating that more than 90% of MS viewed all five videos. As a further recommendation, we suggest implementing a delivery program capable of tracking student viewing times. Such a system would enable more precise observation and interpretation of the outcomes. Moreover, the COVID-19 pandemic necessitated government-imposed social distancing measures, which resulted in individuals being required to stay at home. As a consequence, a considerable number of fifth-year MS engaged in self-study and self-preparation for the NL examination within the confines of their residences, potentially limiting their access to online learning resources. Moreover, the abrupt change form on-site to online learning raised questions among some students regarding the quality and impact of this educational modality. As a result, our study aimed to provide solid data concerning the quality and effectiveness of the online learning system, although the response rate in our study is limited to slightly more than half of all fifth-year MS, which may not fully represent the entire student population.

Nonetheless, one of our study’s strengths is that it is the first perception evaluating cross-sectional study of the online version of IPR among urban MS in our country. We all appreciate the value of on-site learning, as it allows for direct observation of participant engagement and facilitates interpersonal communication. However, the COVID-19 outbreak prompted us to consider alternative options that would best serve our students. In light of our study, we propose assessing positive feedback, both qualitative and quantitative, on online learning as an alternative for students who are unable to attend in-person session due to various reasons such as illness, traffic, flood or living in remote area. This will provide them with more options and opportunities for their education. Our results highlight the need to continue providing online education for MS to summarize and enhance their knowledge in preparation for their examination, together with the well-constructed core pediatric curriculum.

## Conclusions

In summary, limited in-person contact remains a hindrance to quality education among all students, including MS. This study found that the online version offers an alternative to continue providing satisfactory education for MS. Further multicenter studies including all medical topics and evaluating long-term outcome are warranted.

## Data Availability

The datasets used and/or analyzed during the current study are available from the corresponding author on reasonable request.

## Abbreviations

COVID-19: Coronavirus disease 2019
GPA: Grade point average
GPAx: Accumulated grade point average
IPR: Intensive pediatric review course
IQR: Interquartile range
MS: Medical students
NLE: National License Examination;
OIPR: Online intensive pediatric review
OMPE: Online mock pediatric examination
OSCE: Objective structured clinical examination
SD: Standard deviation
